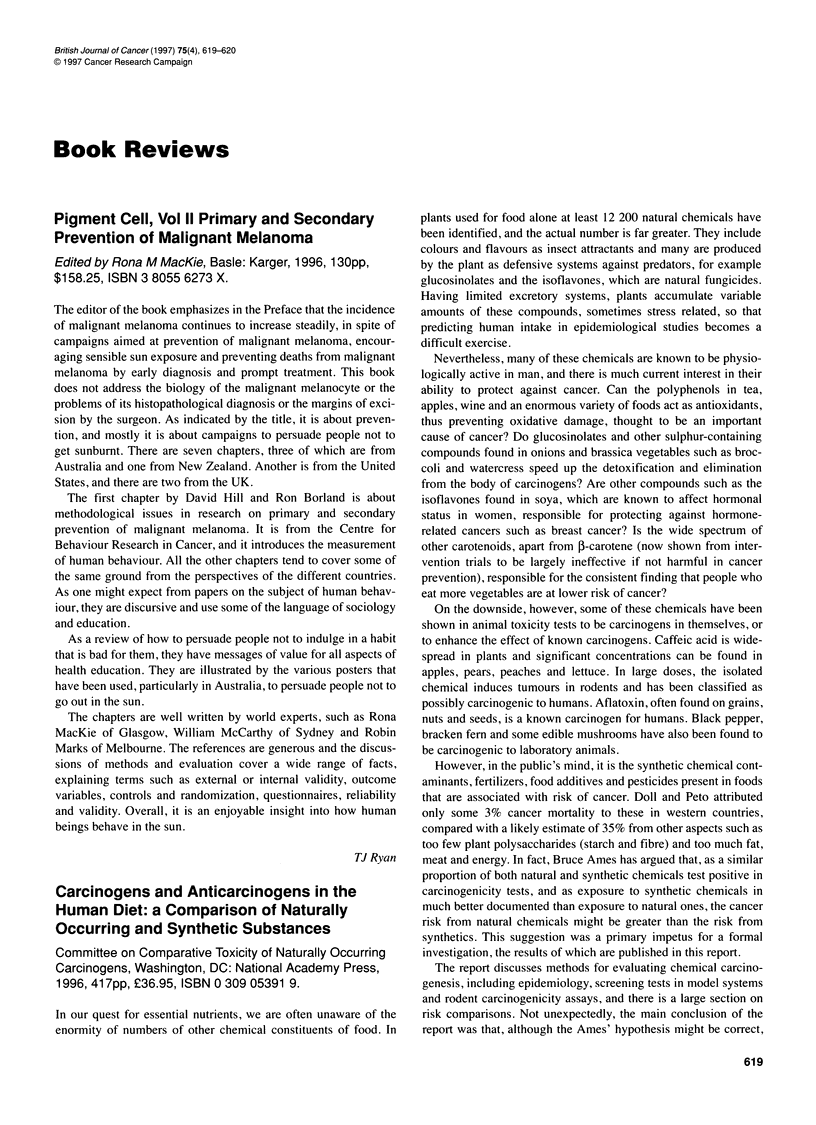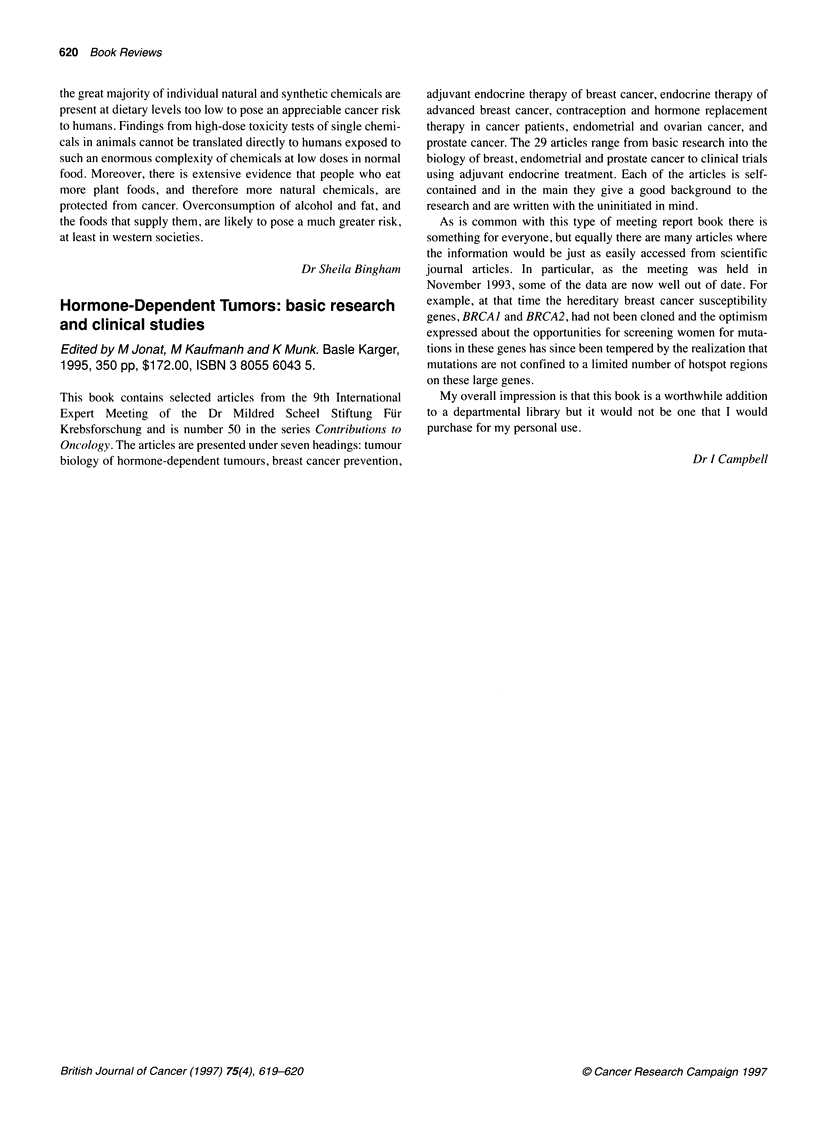# Carcinogens and Anticarcinogens in the Human Diet: a Comparison of Naturally Occurring and Synthetic Substances

**Published:** 1997

**Authors:** Sheila Bingham


### Carcinogens and Anticarcinogens in the Human Diet: a Comparison of Naturally Occurring and Synthetic Substances

Committee on Comparative Toxicity of Naturally Occurring Carcinogens, Washington, DC: National Academy Press, 1996, 417pp, £36.95, ISBN 0 309 05391 9.

In our quest for essential nutrients, we are often unaware of the enormity of numbers of other chemical constituents of food. In plants used for food alone at least 12 200 natural chemicals have been identified, and the actual number is far greater. They include colours and flavours as insect attractants and many are produced by the plant as defensive systems against predators, for example glucosinolates and the isoflavones, which are natural fungicides. Having limited excretory systems, plants accumulate variable amounts of these compounds, sometimes stress related, so that predicting human intake in epidemiological studies becomes a difficult exercise.

Nevertheless, many of these chemicals are known to be physiologically active in man, and there is much current interest in their ability to protect against cancer. Can the polyphenols in tea, apples, wine and an enormous variety of foods act as antioxidants, thus preventing oxidative damage, thought to be an important cause of cancer? Do glucosinolates and other sulphur-containing compounds found in onions and brassica vegetables such as broccoli and watercress speed up the detoxification and elimination from the body of carcinogens? Are other compounds such as the isoflavones found in soya, which are known to affect hormonal status in women, responsible for protecting against hormone-related cancers such as breast cancer? Is the wide spectrum of other carotenoids, apart from β-carotene (now shown from intervention trials to be largely ineffective if not harmful in cancer prevention), responsible for the consistent finding that people who eat more vegetables are at lower risk of cancer?

On the downside, however, some of these chemicals have been shown in animal toxicity tests to be carcinogens in themselves, or to enhance the effect of known carcinogens. Caffeic acid is widespread in plants and significant concentrations can be found in apples, pears, peaches and lettuce. In large doses, the isolated chemical induces tumours in rodents and has been classified as possibly carcinogenic to humans. Aflatoxin, often found on grains, nuts and seeds, is a known carcinogen for humans. Black pepper, bracken fern and some edible mushrooms have also been found to be carcinogenic to laboratory animals.

However, in the public's mind, it is the synthetic chemical contaminants, fertilizers, food additives and pesticides present in foods that are associated with risk of cancer. Doll and Peto attributed only some 3% cancer mortality to these in western countries, compared with a likely estimate of 35% from other aspects such as too few plant polysaccharides (starch and fibre) and too much fat, meat and energy. In fact, Bruce Ames has argued that, as a similar proportion of both natural and synthetic chemicals test positive in carcinogenicity tests, and as exposure to synthetic chemicals in much better documented than exposure to natural ones, the cancer risk from natural chemicals might be greater than the risk from synthetics. This suggestion was a primary impetus for a formal investigation, the results of which are published in this report.

The report discusses methods for evaluating chemical carcinogenesis, including epidemiology, screening tests in model systems and rodent carcinogenicity assays, and there is a large section on risk comparisons. Not unexpectedly, the main conclusion of the report was that, although the Ames' hypothesis might be correct, the great majority of individual natural and synthetic chemicals are present at dietary levels too low to pose an appreciable cancer risk to humans. Findings from high-dose toxicity tests of single chemicals in animals cannot be translated directly to humans exposed to such an enormous complexity of chemicals at low doses in normal food. Moreover, there is extensive evidence that people who eat more plant foods, and therefore more natural chemicals, are protected from cancer. Overconsumption of alcohol and fat, and the foods that supply them, are likely to pose a much greater risk, at least in western societies.

*Dr Sheila Bingham*